# Brain Response to Primary Blast Wave Using Validated Finite Element Models of Human Head and Advanced Combat Helmet

**DOI:** 10.3389/fneur.2013.00088

**Published:** 2013-08-02

**Authors:** Liying Zhang, Rahul Makwana, Sumit Sharma

**Affiliations:** ^1^Department of Biomedical Engineering, Wayne State University, Detroit, MI, USA

**Keywords:** traumatic brain injury, primary blast, finite element model, advanced combat helmet model, human head model, intracranial pressure, brain strain and strain rate, head protection

## Abstract

Blast-induced traumatic brain injury has emerged as a “signature injury” in combat casualty care. Present combat helmets are designed primarily to protect against ballistic and blunt impacts, but the current issue with helmets is protection concerning blasts. In order to delineate the blast wave attenuating capability of the Advanced Combat Helmet (ACH), a finite element (FE) study was undertaken to evaluate the head response against blast loadings with and without helmet using a partially validated FE model of the human head and ACH. Four levels of overpressures (0.27–0.66 MPa) from the Bowen’s lung iso-damage threshold curves were used to simulate blast insults. Effectiveness of the helmet with respect to head orientation was also investigated. The resulting biomechanical responses of the brain to blast threats were compared for human head with and without the helmet. For all Bowen’s cases, the peak intracranial pressures (ICP) in the head ranged from 0.68 to 1.8 MPa in the coup cortical region. ACH was found to mitigate ICP in the head by 10–35%. Helmeted head resulted in 30% lower average peak brain strains and product of strain and strain rate. Among three blast loading directions with ACH, highest reduction in peak ICP (44%) was due to backward blasts whereas the lowest reduction in peak ICP and brain strains was due to forward blast (27%). The biomechanical responses of a human head to primary blast insult exhibited directional sensitivity owing to the different geometry contours and coverage of the helmet construction and asymmetric anatomy of the head. Thus, direction-specific tolerances are needed in helmet design in order to offer omni-directional protection for the human head. The blasts of varying peak overpressures and durations that are believed to produce the same level of lung injury produce different levels of mechanical responses in the brain, and hence “iso-damage” curves for brain injury are likely different than the Bowen curves for lung injury.

## Introduction

Blast-induced traumatic brain injury (bTBI) is a signature injury of the recent wars, affecting a majority of the military casualties (1). According to the Defense Manpower Data Center’s (2) statistics analyzing the Global War on Terrorism casualties from October 7, 2001 through April 4, 2011, explosive weaponry including improvised explosive devices (IEDs), rocket-propelled grenades, and other explosive devices, caused approximately 74% of all killed in action and wounded in action. Department of Defense Armed Forces Health Surveillance Center (AFHSC) reported a total of 229,106 of the service members have been diagnosed with TBI between 2000 and the third quarter of 2011, over 75% of these cases being of mild TBI without any physical or visible brain damage by imaging (3).

Historically, the gas-containing organ systems such as the lungs, the ear, and the bowel were frequently injured from blast insult. Primary blast wave induced TBI is not new. The term “shell shock” was formerly used to describe the brain injury occurring to soldiers in the absence of external wounds in 1940’s (4). In recent military conflicts, the documented incidence of bTBI has been increased as soldiers are survived from non-lethal levels of blast exposure. For head protection, combat helmets remain the primary equipment of protection for the warfighters. Much research has been conducted in order to evaluate ballistic impact response of the modern combat helmets, however presently soldiers are frequently exposed to blast threats due to increased use of improvised explosive weaponry. Blast injuries are categorized by mechanisms into primary, secondary, tertiary, and quaternary injuries (5–8). Soldiers are sustaining injuries through all these different mechanisms. Out of all these types of blast injuries, soldiers are mostly vulnerable to primary and secondary blast insults (9).

The effects of the primary blast wave on brain injury resulting from explosive weaponry have not been addressed in the current helmet designs. Despite prevalence of bTBI, little is known about the biomechanical effects of blast on a human head and how combat helmets affect the blast-induced mechanical loads in the brain. The measurements of wave propagation patterns and stress concentrations within an *in vivo* brain continue to be a significant challenge, due in part to the limitation of physical research methods at such severe loading conditions (high overpressure, sharp rise, short duration). Very limited number of studies reported measured intracranial pressure (ICP) in rats and pig exposed to a blast wave generated in a gas-driven shock tube (10–13). Due to the use of a limited number of sensor (typically only one) within the animal brain in these experimental studies, the complex biomechanical mechanisms taking place in bTBI, including the wave interaction with the head and subsequent energy transmission through various parts of the head and brain could not be fully examined and quantified. Further more, the profound differences in head anatomy; skull thickness; and brain topology between different species influence the wave transformation patterns through various head structures, making it difficult to translate the biomechanical findings from animals to human conditions. Up to now, the interaction of the blast impact with the head and the biomechanical factors leading to the transfer of various forms of shock energy internally have not been demonstrated in *in vivo* conditions.

Finite element (FE) modeling of traumatic events offers a unique means of calculating complex responses within the biological system under many conditions. Many FE head models have been developed and applied to study focal and diffuse brain injury induced by blunt trauma (14–17). More recently the sophisticated FE head model has been utilized to understand the mechanism of concussion by correlating the internal mechanical parameters with pathophysiological and clinical manifestations of mild TBI in blunt impact (18–21). A coupled Lagrangian–Eulerian method is one of the techniques that have been implemented in the FE solvers for simulating an air blast against a structure. Several FE human head models were developed and applied this technique to simulate the blast loading on the head (22–25). Moore et al. (24) used a FE head model showed that direct propagation of blast waves into the brain could occur. Using an ellipsoidal shape representing the human head, Moss et al. (22) did a blast analysis and suggested that flexure of the skull may contribute to the mechanical loading of the brain. In recent years, a handful of studies using FE models reported both protective and unprotective effects of combat helmets against blast loadings (26–29). The study conducted by Nyein et al. (26) revealed that ACH slightly mitigates the intracranial stresses as compared to unprotected head. Moss and King (29) studied the blunt impact response of ACH helmet numerically at one of the impact sites and compared the responses of the different pad systems as well as influence of other factors such as effect of foam material, coating etc. on the Head Injury Criterion (HIC) by using simplified cylindrical impact geometry. Most of these computer models used were either purely descriptive or lacked detailed geometrical and material characteristics of the helmet system. Up to date, none of these head models used were validated against experimentally measured ICP in cadaver head due to blast and blunt loadings.

Our preliminary study using FE head model indicated that mechanisms involved in primary blast-induced TBI may be due to the coupling of stress waves and stress differential in various brain structures (30, 31). The goal of the current study focused on understanding the brain responses to primary blast insult with and without helmet and further extend the current knowledge about effectiveness of ACH in mitigating brain injuries as a result of blast. Their responses were compared to evaluate the effects of military helmet in mitigating blast wave in various conditions. A detailed FE ACH model was first developed and compared against U.S. Army blunt impact experiments. The helmet was then integrated with FE human head that has been recently subjected to validation against cadaveric ICP measurements in shock tube experiments. FE analyses of human head and helmet model subjected to various blast loadings were performed to characterize the resulting biomechanical responses of the brain to blast threats of various conditions.

## Materials and Methods

### Development and validation of 3D FE model of ACH

#### Development of FE models of ACH

**Figure 1:**
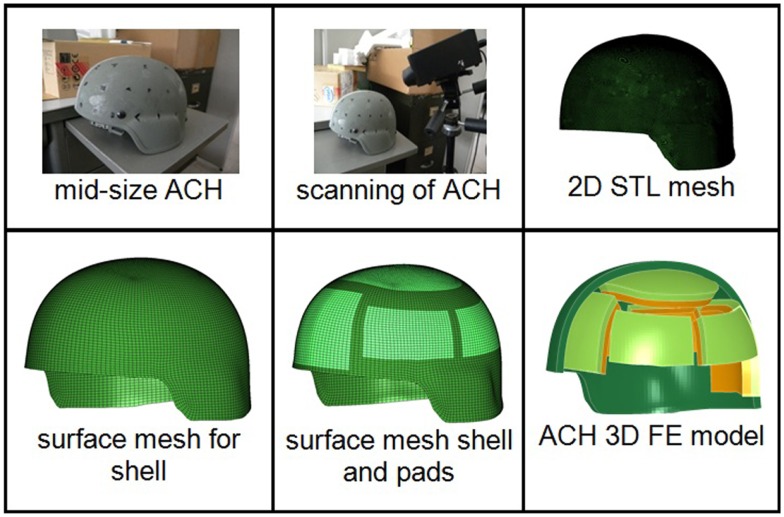
**Step by step procedure for developing a 3D FE ACH model**.

The current Advanced Combat Helmet (ACH) has a 10 mm thick shell with pad system configuration consisting of seven pads in three different shapes. Each 20 mm thick foam is comprised of two parts – hard foam known as the impact liner and soft foam called the comfort liner. An actual mid-size ACH provided by Team Wendy (Cleveland, OH, USA) was used as the prototype. The outer geometry of the helmet shell was obtained by a 3D digital scanner EScan 3363 (3D Digital Corp, CT, USA). The output data from the scanner was 2-D surface mesh in STL format. These 2-D surface meshes were then imported into a mesh pre-processor Hypermesh 10 (Altair Engineering, MI, USA) to generate the multiple surfaces and shell element mesh. The shell element mesh then was extruded inward to the desired thickness of the actual helmet shell using eight-noded brick element meshes (Figure 1). To mesh the seven pads of the ACH helmet, a surface mesh was first generated at the interior of the helmet shell where the pad was attached and the FE nodes were directly connected between the padding and inner shell elements. The sizes of the surface mesh conforming to the actual pad dimensions were then extruded inward to the actual thickness of the pad to create the eight-node brick solid mesh (Figure 1). The pads were separated into two components differentiating the two-part foams with a thickness about 10 mm for each foam. The entire ACH helmet model consisted of over 81,000 elements with an element size of approximately 2–3 mm.

A FE model of a mid-size Department of Transportation (DOT) headform was also built (51) for comparing the blunt impact responses of integrated ACH/Headform assembly against U.S. Army blunt impact experiments on ACH (32).

The helmet outer shell is composed of a Kevlar^®^, a trade name for DuPont’s organic fiber in aromatic polyamide (aramid) family. A composite failure material model MAT_COMOSITE_DAMAGE (MAT_22) available in LS-DYNA (LSTC, Livermore, CA, USA) was chosen to model the woven fabric reinforced aramid laminates of the ACH shell. This material model allows assignment of different material properties to the fibers in three orthogonal directions (a, b, and c). The material axes were properly assigned in the FE model first. Then a transversely isotropic material was assumed for the helmet shell since where one set of moduli and strengths were for the radial directions (a and b) while other ones for the tangential directions (c). A transversely isotropic material was assumed and the failure parameters were assumed to have negligible effect since the shell experienced very small deformation and never approached its failure stresses in the simulations as reported by Moss and King (29). The material parameters for the shell material were based on the data reported in literature (Table 1) (33, 34).

**Table 1:** **Material parameters defined for the shell model**.

Density ρ (kg/mm^3^)	Young’s modulus *Ea* (GPa)	Young’s modulus *Eb* (GPa)	Young’s modulus *Ec* (GPa)	Poisson’s ratio ν*ba*
1.23 × 10^−6^	18.5	18.5	6	0.25
Poisson’s ratio ν*ca*	Poisson’s Ratio ν*cb*	Shear modulus *Gab* (GPa)	Shear modulus *Gbc* (GPa)	Shear modulus *Gca* (GPa)
0.33	0.33	0.77	2.72	2.72

**Figure 2:**
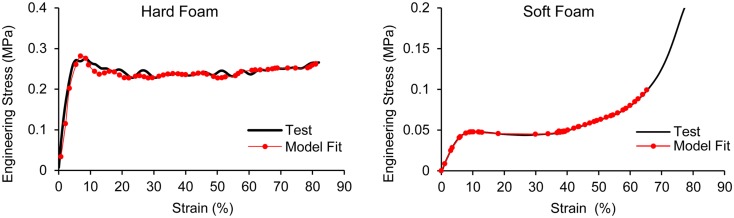
**Stress-strain data and model fit for the hard and soft foams**.

The padding system in the ACH utilizes pads known as Zorbium^®^ Action Pad (ZAP^TM^) manufactured by Team Wendy. Zorbium^®^ is a polyurethane based foam material and with stress-strain behavior that is loading rate dependent. The compressive properties of the hard (density: 6.3 × 10^−8^ kg/mm^3^) and soft foams (density: 6.1 × 10^−8^ kg/mm^3^) were obtained from standard ASTM material testing provided by Team Wendy. The uniaxial compression tests were conducted at strain rates of 0.02, 0.2, 2, 20, and 200 s^−1^ at a normal strain of 80% (Figure 2). The material behaviors of the two foams were modeled by adopting MAT_LOW_DENSITY_FOAM (MAT_57) from the LS-DYNA material library. The model requires uniaxial stress-strain curve, an elastic modulus, and a one-term Prony series describing the reference modulus and time decay constant. The elastic moduli of the hard (8.4 MPa) and soft foams (840 kPa) were used as reference moduli along with a decay constant of 5 ms^−1^. Stress-strain data obtained at the highest compressive strain rate (200 s^−1^) tests performed on the two foams were selected to model high rate blast event.

#### Helmet model blunt impact response comparison

The impact energy attenuation responses of the ACH from a set of laboratory sets were reported by McEntire and Whitley (32). In that study, each ACH helmet was tested at two impact velocities of 10 and 14 feet per second (fps), three environmental conditions and seven impact sites. The seven impact locations were front, rear, left, right, crown, and left and right nape. The maximum and the mean peak resultant acceleration at the center of gravity of the headform (c.g.) were measured for each test. The data from two impact velocities at ambient condition were used to compare the response of the FE ACH helmet model. Similar setup as outlined in the experiments was made with the FE parts of the ACH, headform, and test anvil. In accordance with the helmet impact test configuration, the headform/helmet model movement was constrained to only allow translate in one direction (vertical direction) without rotational motion. The resultant acceleration predicted at the c.g. headform from the FE simulations was compared with that measured experimentally (32).

### Integration of human head model and ACH model

**Figure 3:**
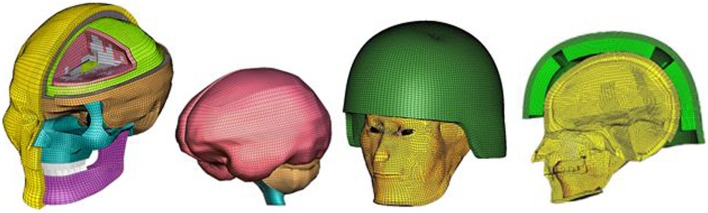
**An anatomically accurate finite element model of the human head (WSUHIM) and the integrated WSUHIM and ACH helmet model**.

The sophisticated FE human head model, Wayne State University Head Injury Model (WSUHIM) previously developed by Zhang et al. (35) was used to capture the intracranial responses from blast loadings with and without ACH helmet in use (Figure 3). This anatomically inspired, high resolution FE model features fine anatomical details of the human head, including the scalp, skull with an outer table, diploë, and inner table, dura, falx cerebri, tentorium, sagittal sinus, transverse sinus, bridging veins, cerebral spinal fluid (CSF), arachnoid membrane, pia mater, hemispheres of the cerebrum with distinct white and gray matter, cerebellum, brainstem, lateral ventricles, third ventricles, facial bones (cortical and spongy bones), nasal cartilage, teeth, temporal mandibular joint, ligaments, flesh, and skin. The entire head model is made up of over 330,000 elements and uses 15 different material properties and constitutive models for various tissues in the head. Most of the biological materials exhibited elastic and viscous properties. Brain tissue is a hydrated soft tissue exhibiting incompressible viscoelastic characteristics (36, 37). Traditionally, the brain material has been approximated by a Kelvin (viscoelastic) model, which is a combination of linear springs and dashpots. The behavior of this material is characterized as viscoelastic in shear with the deviatoric stress rate dependent on the shear relaxation modulus, whereas the hydrostatic behavior of the brain was considered elastic. The shear moduli of the white matter was assumed to be 25% higher than the gray matter due to its fibrous nature. The viscoelastic properties defined for the brain, CSF, and ventricles are shown in Table 2 of the revised manuscript. The bulk modulus for brain tissues, CSF, and ventricles was assumed to be 2.19 GPa. The material properties defined for the other tissues of the head model can be found in the previous publications (20, 35, 38). The model has been subjected to rigorous validation (20, 35) against available experimentally measured ICP, ventricular pressure, brain/skull relative motion, and facial impact responses from cadaveric dynamic impact tests (39–43).

**Table 2:** **Viscoelastic material properties used for brain in the head model**.

Tissues	Shear modulus (kPa)		Decay constant (s^−1^)
	*G*_0_	*G*_∞_	
Gray matter	10	2	80
White matter	12.5	2.5	80
Brainstem	22.5	4.5	80
Cerebellum	10	2	80
CSF/ventricles	1	0.2	80

Recently, the WSUHIM model was applied to head interaction with the blast wave generated from WSU shock tube (31). The model predicted ICP at four locations within the brain were partially validated against the experimentally measured cadaveric data subjected to blast insults of different intensities (74–104 kPa) (44). The model predictions showed reasonably correlation with the experimental results in terms of trends in frontal, parietal, occipital, and ventricular regions which has never been reported previously for any computer head model subjected to blast loadings (31, 44). This human head model was utilized and integrated with the ACH model developed in this study to understand the biomechanical responses involved within the brain during a simulated variety of blast loading conditions occurring in open field environments. A surface to surface contact definition with a coefficient of friction of 0.4 was defined between the head and helmet paddings with the positioning of the ACH according to basic guidelines and instructions in the ACH technical manual (45) (Figure 3).

### Intracranial response to blast loadings with and without helmet in use

#### Open field blast simulation based on Bowen’s curve

The levels of overpressure and associated pulse duration of a forward-facing blast were selected based on Bowen’s iso-lung damage curves, which estimate lung tolerance to free field blast at sea level for a 70 kg unarmored human (46). Four levels of peak overpressures ranging from 0.27 to 0.66 MPa and durations from 1 to 3 ms were selected to simulate the blast wave in open field scenarios. Table 3 lists the net weight of TNT explosives and the stand-off distances determined for the four cases (Cases 1–4) using the scaling equation (47). These levels of overpressures had been utilized and verified in our previous study to characterize blast wave interaction with the head model and subsequent intracranial responses (30). The models of both the head and helmeted head were positioned forward with respect to the center of the TNT explosion according to the various stand-off distances.

**Table 3:** **Stand-off distances and explosives required to achieve the overpressure for four blast loading cases**.

Cases	Duration (ms)	Peak overpressure (MPa)	TNT weight (kg)	Stand-off distance (m)
1	1.0	0.66	0.85	1.06
2	1.5	0.46	1.5	1.45
3	2.0	0.35	1.7	1.85
4	3.0	0.27	5.4	2.80

**Figure 4:**
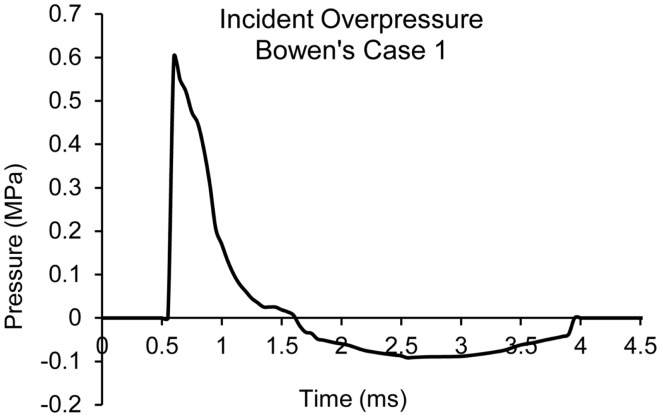
**Plot of blast pressure time history in the vicinity of the head for Bowen’s Case 1**.

The FE models of TNT and air and their material property definitions were the same as those utilized in the previous study (30). The detonation and expansion of the TNT explosive materials were described using the Jones–Wilkins-Lee (JWL) equation of state (EOS) along with a high explosive material property definition. The JWL equation is described as:

$$p=A\left(1-\frac{\omega }{R_{1}V}\right)e^{-R_{1}V}+B\left(1-\frac{\omega }{R_{2}V}\right)e^{-R_{2}V}+\frac{\omega E}{V}$$

where *V is relative volume*. *E* is specific internal energy. *A, B, R_1_, R_2_*, ω are JWL fitting parameters. The parameters chosen were based on literature (48). The blast wave propagation in air, interaction with the head model, and the subsequent structural response in the brain as the pressure wave coupled with various anatomical structures were simulated using the coupled multi-material Lagrangian–Eulerian, fluid-structural interface (FSI), and Lagrangian method in LS-DYNA 971. Figure 4 shows the plot of a blast pressure time history for one of the Bowen’s cases (Case 1) in the vicinity of where the head would be in the blast simulation space.

#### Brain response comparison between the head with and without helmet

The biomechanical response parameters within the brain tissues, including the ICP, maximum principal strain (ε), the rate of the maximum principal strain change (*d*ε*/dt*), and the product of strain and strain rate [(ε)•(dε∕dt)] were computed and analyzed in terms of the response time histories and peak magnitudes to assess the likelihood of brain injury potential in given loading conditions. These tissue-level injury predictors and associated threshold limits applied here were those previously proposed as relevant biomechanical parameters for coup-contrecoup injury and mild traumatic brain injury (mTBI) from blunt impact events (19–21, 49). These injury predictors were applied in this study to measure the potentials of tissue-level damage as predicted by the model in blast loading environment. The anatomical brain locations, including the frontal, temporal, and occipital cerebrum regions along with the midbrain and brainstem were monitored and compared between different cases for both the models with and without the helmet.

#### Effects of head orientation on brain responses with and without helmet

Depending on the head orientations in relation to the oncoming wave direction, the asymmetric human head may experience non-uniform response to blast insult of same severity. Additionally, whether the current ACH helmet design offers equal protection to the head was of concern. In the current study, three different head orientations with respect to the oncoming wave propagation direction were simulated and compared. The helmeted head model were subjected to sideways blast (lateral-to-lateral axis) (Case 5) and backwards (posterio-anterior axis) (Case 6) blast directions and the resulting brain responses were compared to that of a helmeted head model subjected to forward blast condition (Case 3).

## Results

### Comparison of ACH model against blunt impact tests

**Figure 5:**
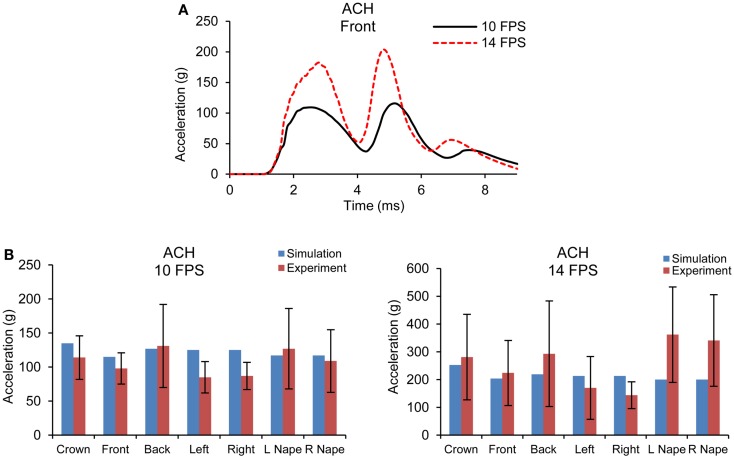
**(A)** Acceleration time traces predicted by FE ACH/headform model from blunt impacts at 10 and 14 fps **(B)** Comparison of peak acceleration at the c.g. of the headform between FE model predictions and experiments at 10 and 14 fps.

Figure 5A shows the acceleration time traces of the headform at 10 and 14 fps for one of the impact sites simulated for the ACH/headform. Figure 5B shows the comparison of peak head acceleration magnitudes between the model simulations and experiments for all impact locations. It is observed that model predicted accelerations at higher velocity impact did not increase as drastically as observed in the experiments. For all the impact locations, the overall accelerations predicted by the FE model were found within the range of experimental results.

### Brain response comparison between the head with and without ACH helmet

#### Intracranial pressure

**Figure 6:**
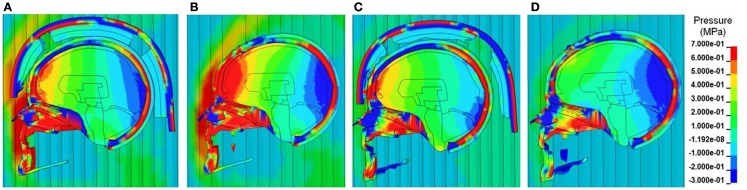
**Pressure contours across the face, scalp, skull and various intracranial components in the midsagittal plane at two selected time points (A, B) T = 2 ms and (C, D) T = 3 ms, when the blast wave interacting with the helmeted head (A, C) and non-helmeted head (B, D) (Case 3 blast loading condition)**.

Figure 6 shows the comparison of the pressure contours at different times across different layers of the head structures between the cases with and without ACH following detonation of explosive for one of the Bowen’s cases (Case 3). As depicted in the sagittal sections, both positive and negative stress wave responses were more profound in the brain without a helmet as compared to those in the helmeted head. In the case without the helmet, at about 2 ms the pressure wave directly impinged on the scalp, propagated through the skull then coupled with the brain at the coup and various regions in the brain (Figure 6B). The ICP was highest at the frontal lobe. In the case of with helmet (Figure 6A), the ACH shell and paddings attenuated some of blast wave from direct entry to the head, resulting in stress wave reduction across the head and inside the cranial cavity, particularly in the coup region underneath the paddings. In the meantime, partial blast waves directly entered through the frontal rim of the helmet and the gap between the helmet and head causing a significant amount of foam deformation. This suggested that some of the wave energy were absorbed by foam compression while the blast wave entering through the gap might directly transmit to the head. However, the overall ICP responses due to the initial stage of loading (2 ms) were reduced for helmet head cases. At a later stage (3 ms after detonation), in the case without helmet (Figure 6D), a majority of the compressive stress waves dissipated in most of brain region, except in the occipital lobe some negative pressure spikes with similar magnitude to the initial negative pressure was observed. It was likely due to a complex wave propagated from the skull that further interacted with the fluid CSF and brain tissue interfaces. In the case with helmet (Figure 6C), due to the tight coupling of the head with the helmet, the padding system acted as a transmission pathway for the blast waves transmitted through the skull to the brain tissue. It was observed that the ACH shell pushed onto the head resulting in second stage loading of the head due to movement of the helmet onto the head. As a result, the coup brain sustained a second positive stress similar to the magnitude of the ICP at 2 ms induced by transmitted blast wave.

**Figure 7:**
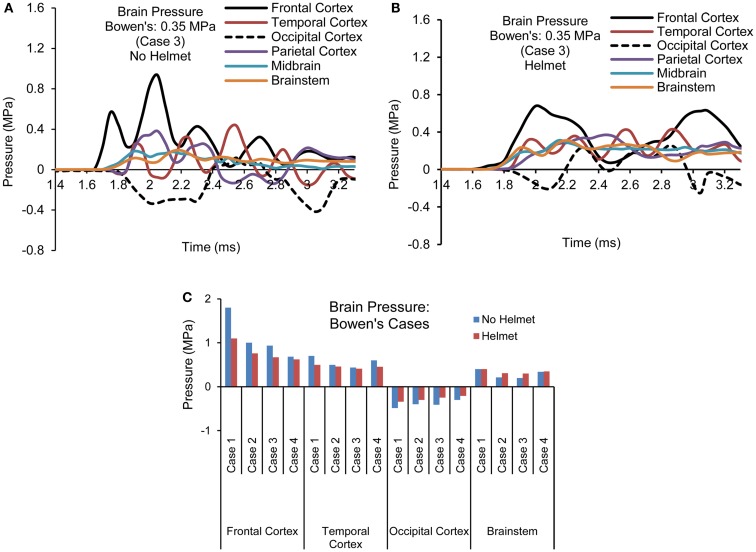
**Pressure time histories predicted in the brain of various regions (Case 3) (A) No helmet (B) Helmet, and (C) Comparison of peak pressure for all four cases based on Bowen’s curves**.

Figures 7A,B show the pressure time histories predicted by the two models at various cortical regions, midbrain, and lower brainstem of the brain for blast loading Case 3. Comparing the pressure curve patterns between the two models, the magnitudes and initial rise time appeared to be affected by the presence of the helmet, most notably at the coup and contrecoup sites showing two major peaks due to two stages of loading on the head. Both peak positive and negative pressures were reduced to 0.67 from 0.93 MPa and from −0.41 to −0.26 MPa. Additionally, the rise times of the ICP responses in the cortical regions were slower compared to the cases without the helmet. The overall rate of the pressure change was reduced by 20–40% for the cortical regions but remained similar for the midbrain and brainstem regions between the helmeted and non-helmeted head.

For all the four cases simulated, the temporal and spatial patterns of the pressure responses predicted within the intracranial cavity for the head and helmeted head showed similar trends but the magnitudes of pressures sustained at various regions of the brain varied depending on the blast loading severities between the cases for both models (Figure 7C). As the side-on overpressure increased from 0.27 to 0.66 MPa, the peak coup pressure increased from 0.68 to 1.8 MPa for non-helmeted head and from 0.62 to 1.1 MPa for helmeted head, which showed an average of 36% reduction. The highest negative pressures in the head model varied from −0.3 to −0.48 MPa. In the helmet model, the pressure at these regions ranged from −0.21 to −0.34 MPa suggesting a decrease of around 30% as compared to head model. However, for the midbrain and lower brainstem regions, only minimal changes (about 5%) in peak pressure were found between the two models.

#### Brain strain and strain rate

**Figure 8:**
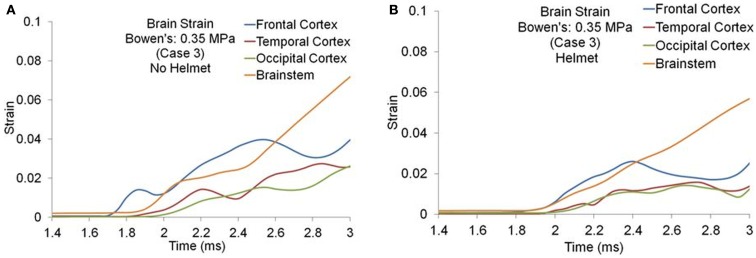
**Strain time histories predicted in the brain of various regions (Case 3) (A) No helmet (B) Helmet**.

**Figure 9:**
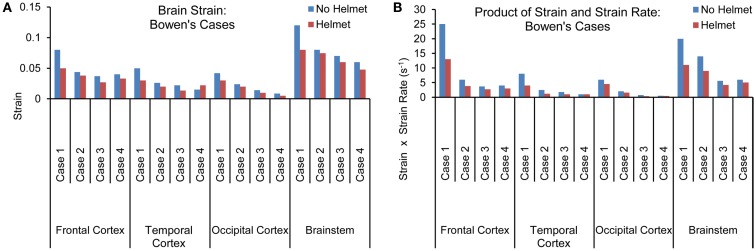
**(A)** Peak magnitudes of maximum principal strain **(B)** Product of strain and strain rate comparison at various brain regions between the models with and without helmet as a result of blast loadings of various severities.

Figures 8A,B show the maximum principal strain (ε) time histories predicted by the two models for one of the Bowen’s cases (Case 3). Figure 9A compares the peak magnitudes of the maximum principal strain sustained in the brain at various regions between the heads with and without helmet in all four cases. In the head model, the strain responses ranged from 0.02 to 0.12 at various cortical and brainstem regions while in the helmeted head, these values ranged from 0.005 to 0.08. Overall, wearing a helmet reduced the strain in the brain by 16 to 30% depending on the loading severities and regions. It was found that the helmet resulted in more reduction in brain strain for a blast insult of higher overpressure with shorter duration pulse (Case 1) as compared to that with its counterparts (Cases 2–4). The strains measured in the brainstem were the highest compared to other regions of the hemisphere through all loading severities for models both with and without helmet in use.

The strain rate, *d*ε(*t*)*/dt* was the temporal strain derivative taken for the elements having the highest strain measures. The product of strain and strain rate, (ε)•(*d*ε(*t*)*/dt*) was then calculated and compared between different regions and two models for all four cases as shown in Figure 9B. Similar to the strain response, the brainstem region sustained the highest (ε)•(*d*ε(*t*)*/dt*) for all three cases except for Case 1, where the highest (ε)•(*d*ε(*t*)*/dt*) was located in the coup site. In comparison to non-helmeted head, the (ε)•(*d*ε(*t*)*/dt*) experienced by the brain at various regions was reduced by an average of 20% among Cases 2–4. For Case 1, (ε)•(*d*ε(*t*)*/dt*) was reduced by 40%. The highest overall (ε)•(*d*ε(*t*)*/dt*) was 24 s^−1^ for the non-helmeted head as compared to below 13 s^−1^ for all helmeted heads for all four cases.

### Effect of head orientation on brain responses

#### Intracranial pressure

**Figure 10:**
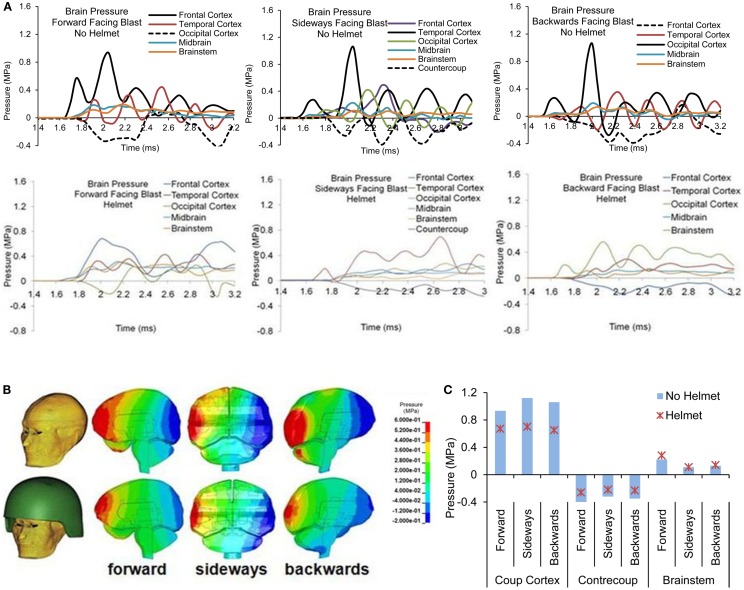
**(A)** Pressure time histories at various regions of the brain in head with and without helmet due to forward, sideways and backwards blast **(B)** Pressure contours across the brain at the time when the pressure exhibits the coup and contrecoup phenomenon, and **(C)** Comparison of the highest positive and lowest negative pressure and brainstem pressures.

Figures 10A–C show the pressure time histories at various cortical regions and comparison of spatial patterns of peak pressure developed across the brain due to blast loadings from three directions between the two models. For both head models, with and without helmet, brain pressure patterns exhibited coup and contrecoup phenomena in which the peak positive pressures occurred at the site facing the direction of the blast wave, whereas the peak negative pressure developed on the region directly opposite to the primary loading site. The peak coup pressures in the head resulting from forward, sideways and backwards blast were 0.93 MPa in the frontal cortex, 1.12 MPa in the temporal cortex, and 1.06 MPa in the occipital cortex, respectively, whereas in the helmeted head model, the pressure was reduced progressively by 27% (forward blast), 37% (sideways blast), and 44% (backward blast). The peak countercoup pressures, i.e., to the occipital, contralateral, and frontal cortex regions, resulting from the three aforementioned blast directions were also found to be reduced by 20–38%. The pressure at the brainstem regions in the non-helmeted head model varied from 0.1 to 0.23 MPa, whereas in the helmeted model, the pressure varied from 0.1 to 0.28 MPa. It was noted that there was no reduction of pressure in the brainstem region. Instead, there was an increase of about 29 and 7% in cases of forward and backwards blast, respectively as a result of wearing the helmet.

#### Brain strain and strain rate

**Figure 11:**
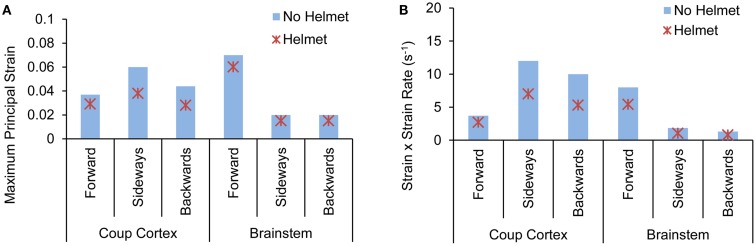
**Comparison of (A) peak maximum principal strain (B) product of strain and strain rate predicted at various brain regions between the head with-without helmet from the forward, sideways and backwards blasts**.

Forward blast resulted in the highest strain in the brainstem (0.07) from both models with and without the helmet. In the head model without helmet, cortical strain was the highest in the temporal cortex (0.06) due to sideways blast whereas backwards blast resulted in the highest strain in the occipital cortex (0.04). With helmet, the brain strain measured at these locations showed an average 25% reduction with the highest reduction of 41% occurring in the occipital region in backwards blast (Figure 11A).

The product of strain and strain rate, (ε)•(*d*ε(*t*)*/dt*) appeared to be in line with the strain responses in terms of relative severities and directional sensitivities exhibited by the two models. In the head model without the helmet, the highest (ε)•(*d*ε(*t*)*/dt*) of 11 s^−1^ occurred in the temporal cortex (coup) due to sideways blast followed by 9 and 7 s^−1^ in the occipital and brainstem regions, respectively. In the helmeted head, the highest (ε)•(*d*ε(*t*)*/dt*) reduction was in the occipital cortex (43%) due to backwards blast followed by 41% reduction in the brainstem region due to sideways blast (Figure 11B).

## Discussion

This communication reports a study conducted in order to understand the biomechanical effects of blasts on human brain and how protective equipment such as the ACH helmet affects the internal brain response parameters to various blast insults in an open field environment. A sophisticated, anatomically detailed, partially validated computer model of a human head coupled with an ACH model was applied successfully to simulate the blast events of varying intensities and directions. The FE models incorporating a hybrid Lagrangian–Eulerian algorithm approach appeared to be useful tools for simulating blast wave phenomena, the interaction of the blast wave with the head and the subsequent internal brain responses to blast loads (30, 31). The computational analysis of such events allows monitoring of the biomechanical response parameters, such as ICP, brain strain, and brain strain rate at any given time and throughout any region/structure of the brain, a task which is technically difficult to perform experimentally in an *in vivo* condition.

The existing human injury tolerance to primary air blast is based on a function of the peak overpressure and positive duration applied to the biological system, such as Bowen’s lung injury curves (46). The head is a very complex structure in which to initiate wave propagation – there are marked differences in densities, material properties, and propagation velocities within various extra- and intracranial tissues, in addition to complex geometrical configurations. Thus, the shock wave, upon interacting with the head, is likely to excite a broad spectrum of frequencies and resulting in stress concentrations in various parts of brain. In the current study, the model showed that the cerebral cortex sustained the highest compressive and tensile stress whereas the central region of brain experienced the greatest strain, and product of strain and strain rate responses. In addition, when subjected to a set of equal blast threats defined using Bowen’s iso-lung threshold curve, the model predicted biomechanical response parameters varying from case to case, suggesting differing severities of brain damage. Among four cases taken from Bowen’s curve, the blast loadings with higher peak overpressure levels/shorter pulse durations produced a higher ICP and brain strain as compared to the brain responses induced by lower peak overpressure/longer pulse duration cases. This may imply that the elastic stress response rather than the viscous response dominated brain behavior in responding to blasts of short pulse duration i.e., between 1 and 3 ms. However, to fully understand the role of viscoelastic effects on brain response to a wide range of blast threats, the application of the blast overpressure with a longer duration pulse (>5 ms) needs to be simulated in the future.

Overall, ACH was found to provide some protection/mitigation against blast resulting in lower ICP, brain strains, and product of strain and strain rate as compared to the cases without the helmet. Nyein et al. (26) in their study had also shown the effectiveness of ACH in reducing intracranial responses in forward blast direction, whereas the present study included the analyses of the brain responses due to blast loadings in forward, sideways, and backward directions. It was observed that a small portion of the blast wave could directly enter through the gap between the helmet and the forehead causing additional deformation on the pads as well as exerting blast loading directly on the head. Comparison of brain response parameters for all cases revealed that the effect of helmet on mitigating the brain responses was effective for brain cortical regions but not for the deep brain structures such as the midbrain/brainstem. Instead, there were some adverse effects on the pressure in the brainstem region due to the use of the helmet for all the forward blast cases. It was also noticed that with helmet, average reduction in brain pressure was the highest (30%) for Case 1 and the lowest (15%) for Case 4. This suggested that the current ACH helmet is more effective in mitigating the blast wave induced by relatively higher intensity-shorter duration pulses than the lower intensity-longer duration pulses.

The effects of the head orientation on the internal brain responses were compared under a given blast dose for both helmeted and non-helmeted heads. Without helmet, sideways blast produced the highest coup and contrecoup responses in comparison with forward and backwards head orientations. The role that loading direction played on severity of injury risk from blast agreed with the findings derived from model analysis of impact-induced TBI (38). With helmet, the helmet showed maximum protection in backwards blast followed by sideways with the least protective effect in frontal blast. It is presumably related to the relatively larger surface area on the back of the helmet than the side and front which may add increased protection by reflecting blast and reducing wave transmission to the head.

The injury risk based on the internal tissue response parameters were assessed using the tissue-level thresholds proposed for blunt impact-induced TBI reported in the literature. The ICP sustained by the brain from a given range of blast insults simulated in this study exceeded the pressure threshold of 235 kPa proposed for contusive injury (50) and 90 kPa for mTBI or concussion (19). However, these proposed pressure limits were derived based on the traumatic event of a longer duration (>4–20 ms or longer). The brain strain levels predicted from the current study were in the subconcussive range. However, the level of product of strain and strain rate indicated 0–25% of probability of sustaining a mild TBI or concussion for some of the blast cases (18, 20, 49). Based on the overall responses experienced by the brain, the current study revealed that the risk of blast-induced brain injury should be evaluated by resulting internal brain response parameters not by the input blast overpressure or its function to the head.

Some of the limitations of the present study may affect the injury assessment of primary blast causing TBI. These include the modeling of a spherical air burst as opposed to a hemispherical ground burst which may close to the shape of the typical IED encounter. Secondly, due to the lack of available blast validation data for ACH helmet, a best effort was made to represent an accurate FE model of ACH model by validating the model responses against experimental drop impact tests. Thirdly, due to the lack of available material properties of the foam and brain tissue characterized at blast loading rates, the results derived from the current model could overpredict brain strain responses and underpredict the strain rate responses, since presumably both foam and brain tissue are rate dependent at higher-rate. Lastly the current model was partially validated for ICP measured in cadaveric heads subjected to blast in shock tube. The open field blast experiments from various loading conditions are required to rigorously validate the computer head model. Future work should include the parametric studies addressing the sensitivities of the material properties of both brain tissues and helmet padding materials under higher-rate loading (>200 s^−1^) in order to evaluate the effect of the material behaviors on resulting brain responses subjected to blast insults.

## Conclusion

The model results revealed that regional and directional variability of the brain stress/strain to various blast environments were the consequence of complex geometry of the head/brain structures and interfaces. The blast input threats defined from Bowen’s iso-lung threshold curve produced dissimilar levels of pressure/stress response in the brain between four cases. The different tissue response could predict potential multi-level damage outcomes rather than the same risk potential as suggested by the iso tolerance curve. A tolerance curve determined specifically for blast-induced neurotrauma is called for.

The current work which extended the study reported in literature (26), provided a comprehensive analysis on the blast mitigation capability of the present military combat helmet with respect to blasts from different directions to the head. Based on the cases studied, blast-induced ICP levels in the helmeted head generally exceed thresholds proposed for contusive brain injury induced by blunt impact. The brain strain and product of strain and strain rate associated with blast loadings were below the thresholds for mild TBI produced by helmeted blunt trauma. The head orientation-dependent responses predicted by the model suggested that directional-specific tolerance criteria are needed for use in helmet design in order to offer omni-directional protection for the human head.

Future work should also incorporate animal experiments with simulations of blast injury to establish correlates between the tissue-level mechanical responses and pathophysiological outcomes following TBI. Such defined tissue-level threshold information, once translated to the human head model, will improve the predictive power of computer models, thus enabling the use of the models as design tools to provide warfighters with improved protection equipment for combating brain trauma.

## Conflict of Interest Statement

The authors declare that the research was conducted in the absence of any commercial or financial relationships that could be construed as a potential conflict of interest.
